# Searching for Small Molecules as Antibacterials: Non-Cytotoxic Diarylureas Analogues of Triclocarban

**DOI:** 10.3390/antibiotics10020204

**Published:** 2021-02-19

**Authors:** Alessia Catalano, Domenico Iacopetta, Antonio Rosato, Lara Salvagno, Jessica Ceramella, Francesca Longo, Maria Stefania Sinicropi, Carlo Franchini

**Affiliations:** 1 Department of Pharmacy-Drug Sciences, University of Bari “Aldo Moro”, 70126 Bari, Italy; antonio.rosato@uniba.it (A.R.); lara.salvagno89@gmail.com (L.S.); f.longo21@studenti.uniba.it (F.L.); carlo.franchini@uniba.it (C.F.); 2 Department of Pharmacy, Health and Nutritional Sciences, University of Calabria, 87036 Arcavacata, Italy; domenico.iacopetta@unical.it (D.I.); jessicaceramella@gmail.com (J.C.); s.sinicropi@unical.it (M.S.S.)

**Keywords:** antibacterial activity, triclocarban, cytoxicity, diarylureas, small molecules, *Staphylococcus aureus*, *Enterococcus faecalis*

## Abstract

Triclocarban (TCC), a broad-spectrum lipophilic antimicrobial agent, is a diarylurea derivative that has been used for more than 60 years as a major ingredient of toys, clothing, food packaging materials, food industry floors, medical supplies and especially of personal care products, such as soaps, toothpaste and shampoo. In September 2016, the U.S. FDA banned nineteen antimicrobial ingredients, including TCC, in over-the-counter consumer antiseptic wash products, due to their toxicity. Withdrawal of TCC has prompted efforts to search for new antimicrobial compounds. In this paper, we present the synthesis and biological evaluation, as antibiotic and non-cytotoxic agents, of a series of diarylureas, analogues of TCC. These compounds are characterized by an intriguingly simple chemistry and can be easily synthesized. Among the synthesized compounds, **1ab** and **1bc** emerge as the most interesting compounds as they show the same activity of TCC (MIC = 16 µg/mL) against *Staphylococcus aureus,* and a higher activity than TCC against *Enterococcus faecalis* (MIC = 32 µg/mL versus MIC = 64 µg/mL). Moreover, **1ab** and **1bc** show no cytotoxicity towards the human mammary epithelial cells MCF-10A and embryonic kidney epithelial cells Hek-293, in opposition to TCC, which exhibits a marked cytotoxicity on the same cell lines and shows a good antitumor activity on a panel of cell lines tested.

## 1. Introduction

**Figure 1:**
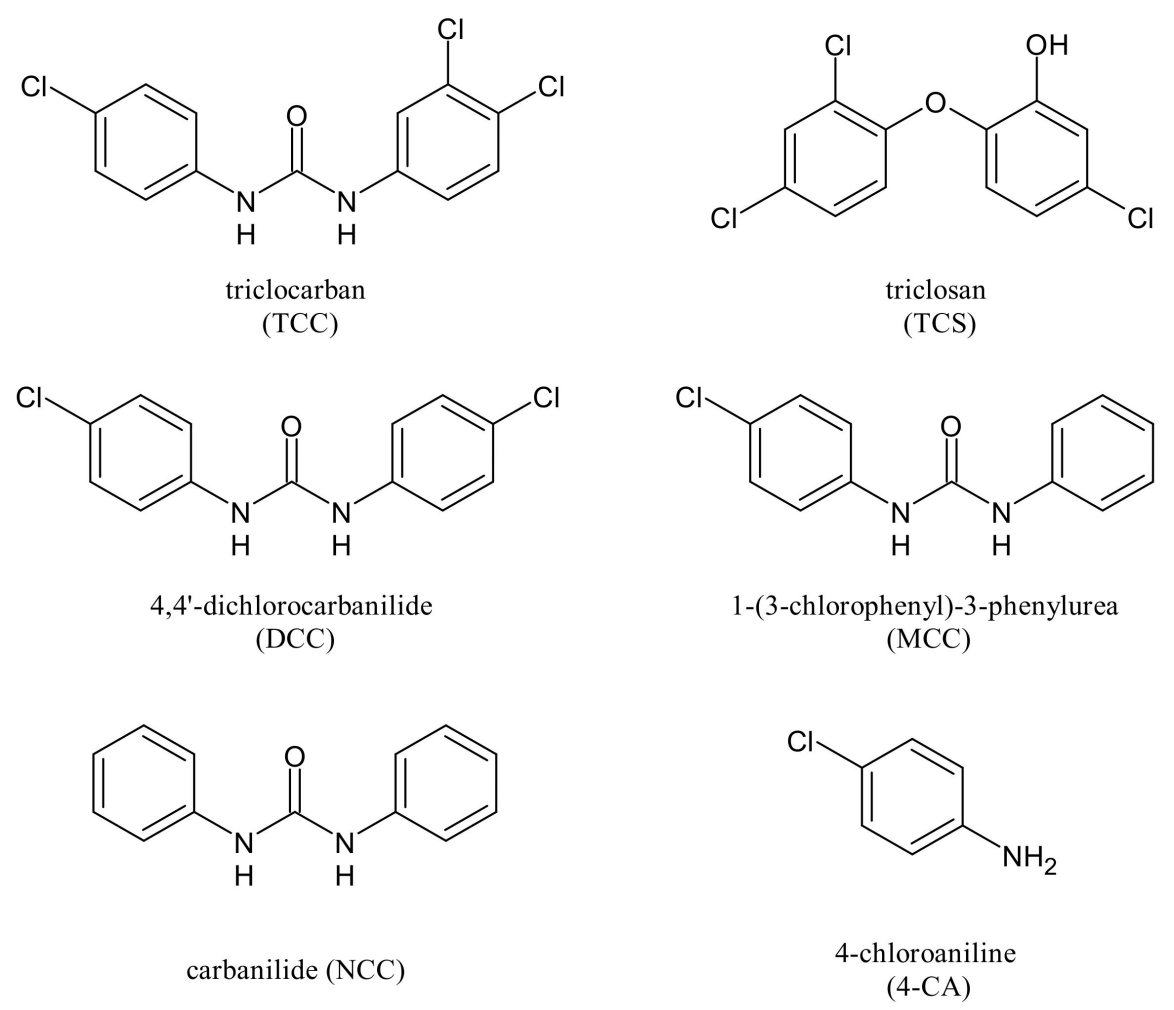
Triclocarban (TCC), triclosan and TCC transformation products.

The polychlorinated aromatic antimicrobials triclocarban (TCC) and triclosan (TCS, Figure 1) are organic compounds that have been widely used in a vast range of applications [1,2]. Triclocarban, *N*-(4-chlorophenyl)-*N’*-(3,4-dichlorophenyl)urea, is a highly effective and broad spectrum antimicrobial that has been successfully used in personal care products for over 60 years [3]. It belongs to the privileged class of diarylureas, which have been recently extensively reviewed for their multiple actions, including the anticancer [4], and have also been proposed for a repositioning as antimicrobial agents and/or for the treatment of new pandemics, including COVID-19 [5,6]. It is predominantly found in household necessities and personal care products, especially dermal cleaning products such as antibacterial bar/liquid soap, body lotion, deodorants, aftershave soaps, hand sanitizers, toothpaste, handwash and mouthwash [7]. Its concentration in the products can be as high as 1.5% [8]. It has been used for a long time to prevent food spoilage and infections because it perturbs microbial fatty acid synthesis and membrane formation [9]. TCC can be dechlorinated to 4,4′-dichlorocarbanilide (DCC), 1-(3-chlorophenyl)-3-phenylurea (MCC) and carbanilide (NCC), or either biologically or abiotically into 4-chloroaniline (4-CA) (Figure 1) [10]. Although TCC has been widely used for over 50 years, only recently some concerns about its endocrine disruptive properties were raised [11,12]. As a halogenated hydrocarbon, TCC is hardly biodegradable, and many studies have reported the occurrence of TCC in wastewater effluent, surface water, biosolid, sediment and soil [13]. Risks of TCC in the environment, on plants and animals, humans and microorganisms have been recently extensively described [14]. TCC is ranked in the top 10 Contaminant of Emerging Concern (CEC) occurrence. In September 2016, the U.S. Food and Drug Administration banned its use in over-the-counter hand and body washes [15], but this compound still retained a large market demand [16]. More recent studies are devoted to the removal of TCC and its dechlorinated congeners from soil, given their toxicity [17]. Thus, the desirable properties of next-generation antimicrobials should include broad-spectrum action and high efficacy toward pathogens but low toxicity to humans and should pose no risk of bioaccumulation. Recently, several diarylureas analogues of TCC were reported as antibacterial [18] and antifungal agents [19] bearing pentafluorosulfanyl and trifluoromethylcoumarine groups, respectively. As small molecules, their design should take into account a good oral bioavailability, following the “Lipinski’s rule of drug-likeness”, also named “the Rule of 5” (Ro5) [20]. 

**Figure 2:**
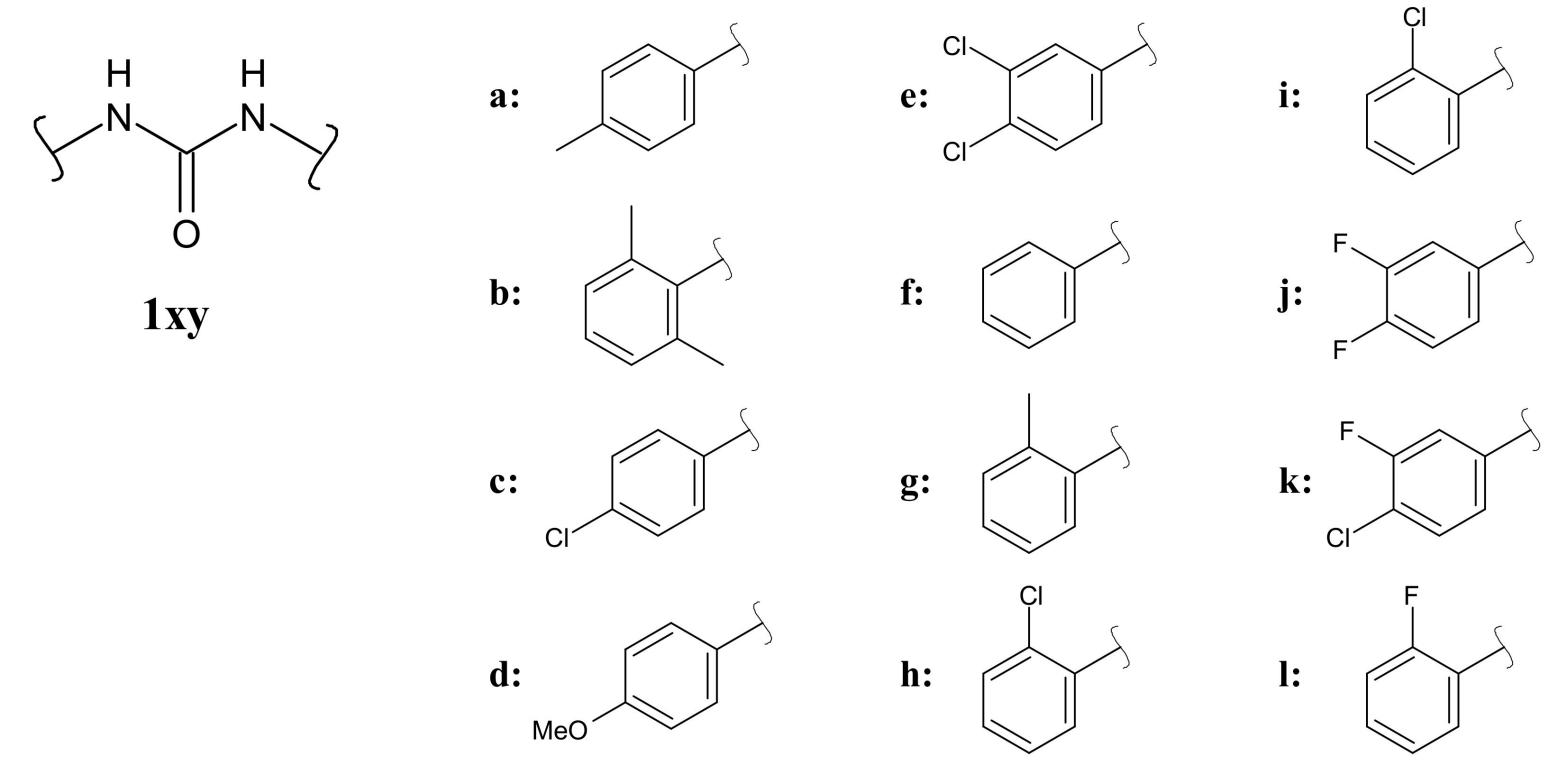
Diarylureas (**a**–**l**) under study.

In this paper, we report the synthesis of a series of diarylureas, particularly diphenylureas or bis-phenylureas (Figure 2), inspired by TCC, bearing different aryl moieties that we have previously studied, such as 2,6-xylyl, *p*-methyl, 3-4-difluoro and others [21,22,23], and their antimicrobial activity evaluation against Gram-positive and Gram-negative bacteria. Moreover, the anticancer effect of TCC (**1ce**) and its active analogues (**1ab** and **1bc**) was evaluated against several human cancer cell lines (breast cancer cells: MCF-7 and MDA-MB-231; uterine cancer cells: HeLa and Ishikawa; melanoma epithelial cells: A2058). Our outcomes clearly showed that TCC possesses a good antitumor activity against the panel of cell lines tested but, it is not selective because of its dramatic cytotoxic effects on normal human epithelial cells, MCF-10A, and embryonic kidney epithelial cells, Hek-293. Alternatively, both **1ab** and **1bc** do not possess any antitumor or cytotoxic activity, at least using the abovementioned cell lines and under our experimental conditions, but they exhibit an antibacterial activity comparable to or even higher than that of TCC. We are confident that these leads could represent promising tools for the development of new effective antibacterial agents and, whenever possible, also compounds with dual activity acting both as antibacterial and anticancer tools [24].

## 2. Results and Discussion

### 2.1. Chemistry

**Scheme 1:**
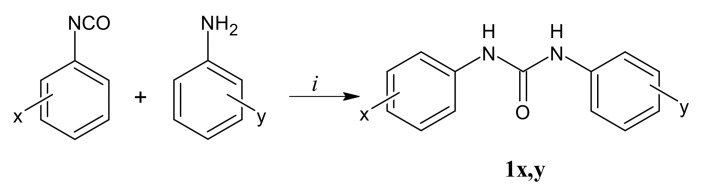
*Reagents and conditions:* (i) acetone, 100 °C, 6 h.

Diphenylureas were easily prepared as depicted in Scheme 1. Final products were obtained by reacting the commercial anilines with the appropriate phenylisocyanate in acetone as reported in the literature [25]. 

### 2.2. Antibacterial Studies

The in vitro Minimum Inhibitory Concentrations (MICs, μg/mL) were determined by the broth microdilution method according to the Clinical Laboratory Standards Institute (CLSI) guidelines. MIC values were recorded as the lowest concentration of compounds at which there was no optically detectable microorganism growth and evaluated by comparing the growth in each well visually with that of the growth in the control well for bacteria. All compounds were dissolved in dimethyl sulfoxide (DMSO) and were tested in a final concentration range of from 512 to 2 µg/mL. MICs for the reference antibiotic norfloxacin against quality control strains were used to confirm the validity of the screen. According to the CLSI guidelines [26], diphenylureas were tested in vitro against a panel of Gram-positive and Gram-negative bacteria belonging to the ATCC collection using the standard microdilution test for the determination of MICs. MICs of the synthesised compounds along with the parent compound TCC and with the standard antibiotic norfloxacin are listed in Table 1. The compounds were also analyzed in terms of molecular mass, log*P* (Table 1), number of hydrogen-bond donors and number of hydrogen-bond acceptors, and most of them obeyed Lipinski’s rule [20]. Our results showed that the highest activity against *S. aureus* was obtained for compounds **1ab, 1bc, 1bd, 1fg, 1bf** and **1ef**, showing MIC values of 16 µg/mL, on a par with TCC. A slightly lower activity was observed for **1ad, 1be, 1gh** and **1gl** (MIC = 32 µg/mL). The other compounds were less active or inactive. Based on our results, it seems that the 3,4-dichloro-disubstitution pattern, which is present in TCC, is not crucial for activity. Out of the six compounds bearing this substitution (TCC versus **1be, 1ef, 1eg, 1ae** and **1de**), only TCC showed high activity. Conversely, compounds **1ab, 1bc, 1bd** and **1bf**, bearing a 2,6-xylyl, showed the highest antimicrobial activity against *Staphylococcus aureus*. Moreover, the activity against *Enterococcus faecalis* was interesting. *E. faecalis* is a ubiquitous member of the healthy human gut microbiota and also a common opportunistic pathogen and leading cause of nosocomial infections [27,28]. It has a high incidence for most infections, and the case numbers have been increasing in recent decades, likely due to its greater ability to persist in healthcare facilities and its higher abundance in the human gut microbiome [29]. Kim et al. (2016) [30] compared the bactericidal effects of plain and antibacterial soap containing 0.3% TCC. The authors found no significative difference between the effects of plain and medicated soap at either temperature, with the only exception of *E. faecalis* ATCC 19433 at 40 °C. **1ab** and **1bc** showed antibacterial activity higher than TCC (MIC = 32 versus 64 µg/mL) against this Gram-positive bacterium.

### 2.3. Cytotoxicity Studies

In order to investigate the anticancer effects of compounds **1ab**, **1bc** and **1ce** (TCC), we used different human cancer cell models, namely, two breast cancer cells (MCF-7 and MDA-MB-231), two uterine cancer cells (HeLa and Ishikawa) and the melanoma epithelial cells A2058. Performing the MTT assay, we determined that compounds **1ab** and **1bc** did not elicit any anticancer effect towards all the cancer cell lines used, at least until the concentration of 200 µg/mL (Table 2). On the contrary, TCC, used as the reference molecule, exerted a strong anticancer effect against the cancer cell lines, with IC_50_ values between 0.64 and 1.68 µg/mL. Unfortunately, TCC also exhibited a marked cytotoxicity on the normal cells used in this assay, the human mammary epithelial cells MCF-10A and embryonic kidney epithelial cells Hek-293, with IC_50_ values of 1.43 ± 0.7 and 1.60 ± 0.8 μg/mL, respectively. Instead, compounds **1ab** and **1bc** did not possess any cytotoxicity towards the same normal cancer cell lines at least below the concentration of 200 µg/mL.

## 3. Materials and Methods

### 3.1. Chemistry

Chemicals were purchased from Sigma-Aldrich or Lancaster. Yields refer to purified products and were not optimized. Compound stuctures were confirmed by ordinary spectrometric analyses (^1^H NMR, ^13^C NMR, LC-MS, IR, Elemental Analysis). Melting points were determined on a Gallenkamp melting point apparatus in open glass capillary tubes and are uncorrected. ^1^H and ^13^C NMR spectra were registered on a Varian VX Mercury spectrometer operating at 300 and 75 MHz for ^1^H and ^13^C, respectively, or an Agilent 500 MHz operating at 500 and 125 MHz for ^1^H and ^13^C, respectively, using DMSO-*d*_6_ as solvent. Chemical shifts are reported in parts per million (ppm) relative to solvent resonance: *δ* = 2.48 ppm (^1^H NMR) and *δ* = 39.9 ppm (^13^C NMR). *J* values are given in Hz. Abbreviations “s-singlet, d-doublet, t-triplet, m-multiplet” are used. Gas chromatography (GC)/mass spectroscopy (MS) was performed on a Hewlett-Packard 6890–5973 MSD at low resolution. Liquid chromatography (LC)/mass spectroscopy (MS) was performed on a spectrometer Agilent 1100 series LC-MSD Trap System VL. The molecular ion was designed as “M^+^”. Elemental analyses were performed on a Eurovector Euro EA 3000 analyzer. The data for C, H, N were within ± 0.4 of theoretical values. 

*1-(2,6-Dimethylphenyl*)-*3-p-tolylurea *(**1ab**) [31]. A solution of 2,6-dimethylphenyl isocyanate (0.26 mL, 1.87 mmol) in acetone RP (4 mL) was added in three portions (in 1.5 h) to a solution of *p*-toluidine (0.20 g, 1.87 mmol) in acetone RP (4 mL). The resulting mixture was refluxed for 6 h. The solid was filtered to give **1ab** as a white solid (0.41 g, 86%), which was recrystallized from CHCl_3_ giving 0.35 g (73%) of white crystals: mp 242–243 °C; GC/MS (70 eV) *m*/*z* (%): 254 (M^+^, 24), 107 (100); IR (KBr): 3288 (NH), 1642 (C=O) cm^−1^; ^1^H NMR (300 MHz, DMSO-*d*_6_): δ 2.18 (s, 6H, 2CH_3_), 2.21 (s, 3H, CH_3_), 6.98–7.10 (m, 5H, Ar), 7.31 (d, *J* = 8.2 Hz, 2H, Ar), 7.63 (br s, 1H, NH, exch. D_2_O), 8.57 (br s, 1H, NH, exch. D_2_O); ^13^C NMR (75 MHz, DMSO-*d*_6_): δ 18.7 (2C), 20.8 (1C), 118.5 (2C), 126.3 (1C), 128.1 (2C), 129.5 (2C), 130.6 (1C), 135.9 (2C), 136.0 (1C), 138.2 (1C), 153.6 (1C). Anal. calcd for C_16_H_18_N_2_O·0.20 H_2_O (257.75)%: C 74.51; H 7.19; N 10.86. Found %: C 74.76; H 7.00; N 10.85.

*1-(2,6-Dimethylphenyl)-3-(4-chlorophenyl)urea *(**1bc**) [32]. Prepared from 4-CA and 2,6-dimethylphenyl isocyanate. Yield: 72% (needle-like white crystals): mp 244–245 °C (CHCl_3_); GC/MS (70 eV) *m*/*z* (%): 274 (M^+^, 13), 127 (100); IR (KBr): 3282 (NH), 1643 (C=O) cm^−1^; ^1^H NMR (300 MHz, DMSO-*d*_6_): δ 2.18 (s, 6H, 2CH_3_), 7.02–7.08 (m, 3H, Ar), 7.27 (d, *J* = 8.8 Hz, 2H, Ar), 7.47 (d, *J* = 8.8 Hz, 2H, Ar) 7.73 (br s, 1H, NH, exch. D_2_O), 8.86 (br s, 1H, NH, exch. D_2_O); ^13^C NMR (125 MHz, DMSO-*d*_6_): δ 18.7 (2C), 119.8 (2C), 125.3 (1C), 126.5 (1C), 128.2 (2C), 129.0 (2C), 135.6 (1C), 136.0 (2C), 139.8 (1C), 153.5 (1C). Anal. calcd for C_15_H_15_ClN_2_O (274.59)%: C 65.57; H 5.50; N 10.20. Found%: C 65.59; H 5.65; N 10.59.

*1-(4-Methoxyphenyl)-3-(p-tolyl)urea *(**1ad**) [33]. Prepared from *p*-toluidine and *p*-methoxyphenyl isocyanate. Yield: 65% (needle-like white crystals): mp 241–242 °C (EtOH 96%); LC/MS (70 eV) *m*/*z* (%): 276 [M^+^, +23]; IR (KBr): 3283 (NH), 1644 (C=O) cm^−1^; ^1^H NMR (300 MHz, DMSO-*d*_6_): δ 2.21 (s, 3H, CH_3_), 3.68 (s, 3H, CH_3_), 6.84 (d, *J* = 8.8 Hz, 2H, Ar), 7.04 (d, *J* = 8.2 Hz, 2H, Ar), 7.26–7.36 (m, 4H, Ar), 8.38 (br s, 1H, NH, exch. D_2_O), 8.43 (br s, 1H, NH, exch. D_2_O); ^13^C NMR (75 MHz, DMSO-*d*_6_): δ 20.8 (1C), 55.6 (1C), 114.4 (2C), 118.6 (2C), 120.4 (2C), 129.6 (2C), 130.8 (1C), 133.3 (1C), 137.7 (1C), 153.2 (1C), 154.9 (1C). Anal. calcd for C_15_H_16_N_2_O_2_·0.33H_2_O (262.12)%: C 68.68; H 6.40; N 10.68. Found%: C 68.37; H 6.16; N 10.55.

*1-(4-Methoxyphenyl)-3-(2,6-dimethylphenyl)urea *(**1bd**) [32]. Prepared from *p*-methoxyphenyl isocyanate and 2,6-dimethylaniline. Yield: 66% (needle-like white crystals): mp 225–226 °C (CHCl_3_); LC/MS (70 eV) *m*/*z* (%): 293 [M^+^, +23]; IR (KBr): 3262 (NH), 1643 (C=O) cm^−1^; ^1^H NMR (300 MHz, DMSO-*d*_6_): δ 2.18 (s, 6H, 2CH_3_), 3.68 (s, 3H, CH_3_), 6.82 (d, *J* = 8.8 Hz, 2H, Ar), 7.03 (s, 2H, Ar), 7.33 (d, *J* = 8.8 Hz, 2H, Ar), 7.59 (s, 1H, Ar) 8.37 (br s, 1H, NH, exch. D_2_O), 8.50 (br s, 1H, NH, exch. D_2_O); ^13^C NMR (75 MHz, DMSO-*d*_6_): δ 18.7 (2C), 55.6 (1C), 114.4 (2C), 120.1 (2C), 120.3 (2C), 126.2 (1C), 128.1 (2C), 133.9 (1C), 135.9 (1C), 153.8 (1C), 154.6 (1C). Anal. calcd for C_16_H_18_N_2_O_2_ (270.33)%: C 71.09; H 6.71; N 10.36. Found %: C 70.70; H 6.58; N 10.35.

*1-(2,6-Dimethylphenyl)-3-(3,4-dichlorophenyl)urea *(**1be**). Prepared from 2,6-dimethylaniline and 3,4-dichlorophenyl isocyanate. Yield: 76% (white crystals): mp 249–250 °C (CHCl_3_/MeOH/Acetone); LC/MS (70 eV) *m*/*z* (%): 331 [M^+^, +23]; IR (KBr): 3301 (NH), 1639 (C=O) cm^−1^; ^1^H NMR (300 MHz, DMSO-*d*_6_): δ 2.17 (s, 6H, 2CH_3_), 7.05 (s, 3H, Ar), 7.31 (d, *J* = 8.8 Hz, 1H, Ar), 7.46 (d, *J* = 8.8 Hz, 1H, Ar), 7.84 (br s, 2H, Ar + NH, exch. D_2_O), 9.06 (br s, 1H, NH, exch. D_2_O); ^13^C NMR (75 MHz, DMSO-*d*_6_): δ 18.6 (2C), 118.5 (1C), 119.5 (1C), 123.0 (1C), 126.7 (1C), 128.2 (2C), 130.9 (1C), 131.4 (1C), 135.3 (1C), 136.1 (2C), 141.0 (1C), 153.4 (1C). Anal. calcd for C_15_H_14_Cl_2_N_2_O·0.50H_2_O (317.05)%: C 56.62; H 4.75; N 8.80. Found%: C 56.78; H 4.43; N 8.93.

*1-(4-Methylphenyl)-3-phenylurea *(**1af**) [34]. Prepared from *p*-toluidine and phenyl isocyanate. Yield: 80% (needle-like white crystals): mp 240–242 °C (CHCl_3_/acetone); LC/MS (70 eV) *m*/*z* (%): 249 [M^+^, +23]; IR (KBr): 3301 (NH), 1634 (C=O) cm^−1^; ^1^H NMR (500 MHz, DMSO-*d*_6_): δ 2.22 (s, 3H, CH_3_) 6.93 (t, *J* = 7.3 Hz, 1H, Ar), 7.06 (d, *J* = 8.3 Hz, 2H, Ar), 7.25 (t, *J* = 7.5 Hz, 2H, Ar), 7.31 (d, *J* = 8.3 Hz, 2H, Ar), 7.42 (d, *J* = 8.3 Hz, 2H, Ar), 8.51 (s, 1H, NH), 8.57 (s, 1H, NH); ^13^C NMR (125 MHz, DMSO-*d*_6_): δ 20.8 (1C), 118.6 (2C), 118.7 (2C), 122.1 (1C), 129.2 (2C), 129.6 (2C), 131.0 (1C), 137.6 (1C), 140.2 (1C), 153.0 (1C).

*1-(2-Methylphenyl)-3-(4-methylphenyl)urea *(**1ag**) [35]. Prepared from *p*-toluidine and *o*-methylphenyl isocyanate. Yield: 74% (white solid): mp 241–242 °C; LC/MS (70 eV) *m*/*z* (%): 263 [M^+^, +23]; IR (KBr): 3305 (NH), 1643 (C=O) cm^−1^; ^1^H NMR (300 MHz, DMSO-*d*_6_): δ 2.21 (s, 6H, 2CH_3_), 6.91 (t, *J* = 7.6 Hz, 1H, Ar), 7.05–7.15 (m, 4H, Ar), 7.32 (d, *J* = 8.2 Hz, 2H, Ar), 7.82 (d overlapping s at 7.83 ppm, *J* = 8.8 Hz, 1H, Ar), 7.83 (s overlapping d at 7.82 ppm, 1H, NH, exch. D_2_O), 8.88 (s, 1H, NH, exch. D_2_O); ^13^C NMR (75 MHz, DMSO-*d*_6_): δ 18.3 (1C), 20.8 (1C), 118.5 (2C), 121.4 (1C), 122.9 (1C), 126.6 (1C), 127.8 (1C), 129.6 (2C), 130.6 (1C), 130.9 (1C), 137.7 (1C), 137.9 (1C), 153.1 (1C). Anal. calcd for C_15_H_16_N_2_O·0.14H_2_O (242.87) %: C 74.18; H 6.76; N 11.53. Found%: C 74.52; H 6.56; N 11.61.

*1-(3,4-Dichlorophenyl)-3-(4-chlorophenyl)urea (***1ce***, TCC)* [36]. Prepared from 3,4-dichloroaniline and *p*-chlorophenyl isocyanate. Yield: 29% (white solid): mp ≥ 250 °C; LC/MS (70 eV) *m*/*z* (%): 317 [M^+^]; IR (KBr): 3296 (NH), 1639 (C=O) cm^−1^; ^1^H NMR (500 MHz, DMSO-*d*_6_): δ 7.31 (d, *J* = 8.8 Hz, 3H, Ar), 7.45–7.50 (m, 3H, Ar), 7.84 (d, *J* = 2.4 Hz, 1H, Ar), 8.92 (s, 1H, NH, exch. D_2_O), 8.99 (s, 1H, NH, exch. D_2_O); ^13^C NMR (125 MHz, DMSO-*d*_6_): δ 118.9 (1C), 119.8 (1C), 120.5 (1C), 123.7 (2C), 126.2 (1C), 129.1 (2C), 131.0 (1C), 131.5 (1C), 138.7 (1C), 140.3 (1C), 152.7 (1C). Anal. calcd for C_13_H_9_Cl_3_N_2_O (315.58) %: C 49.48; H 2.87; N 8.88. Found%: C 49.48; H 3.02; N 8.79.

*1,3-Diphenylurea (***1ff***, NCC)* [37]. Prepared from aniline and phenyl isocyanate. Yield: 77% (white solid): mp 242–243 °C; LC/MS (70 eV) *m*/*z* (%): 235 [M^+^, +23]; IR (KBr): 3328 (NH), 1647 (C=O) cm^−1^; ^1^H NMR (300 MHz, DMSO-*d*_6_): δ 6.95 (t, *J* = 7.3 Hz, 2H, Ar), 7.26 (t, *J* = 7.9 Hz, 4H, Ar) 7.43 (d, *J* = 7.6 Hz, 4H, Ar), 8.65 (s, 2H, NH, exch. D_2_O); ^13^C NMR (75 MHz, DMSO-*d*_6_): δ 118.6 (4C), 122.3 (2C), 129.2 (4C), 140.1 (2C), 152.9 (1C). Anal. calcd for C_13_H_12_N_2_O (212,24)%: C 73.56; H 5.70; N 13.20. Found%: C 73.63; H 5.61; N 13.19.

*1-(2-Methylphenyl)-3-phenylurea* (**1fg**) [33]. Prepared from aniline and *o*-tolyl isocyanate. Yield: 77% (white solid): mp 241–242 °C; LC/MS (70 eV) *m*/*z* (%): 249 [M^+^, +23]; IR (KBr): 3269 (NH), 1631 (C=O) cm^−1^; ^1^H NMR (300 MHz, DMSO-*d*_6_): δ 2.21 (s, 3H, CH_3_), 6.89–6.96 (m, 2H, Ar), 7.09–7.16 (m, 2H, Ar), 7.26 (t, *J* = 7.6 Hz, 2H, Ar), 7.44 (d, *J* = 7.6 Hz, 2H, Ar), 7.81 (d, *J* = 8.2 Hz, 1H, Ar), 7.90 (s, 1H, NH, exch. D_2_O), 9.01 (s, 1H, NH, exch. D_2_O); ^13^C NMR (75 MHz, DMSO-*d*_6_): δ 18.3 (1C), 118.4 (2C), 121.5 (1C), 122.1 (1C), 123.1 (1C), 126.6 (1C), 128.0 (1C), 129.3 (2C), 130.6 (1C), 137.8 (1C), 140.3 (1C), 153.1 (1C). Anal. calcd for C_14_H_14_N_2_O·0.16H_2_O (229.27)%: C 73.34; H 6.30; N 12.22. Found%: C 73.65; H 6.08; N 12.27.

*1-(2,6-Dimethylphenyl)-3-phenylurea *(**1bf**) [32]. Prepared from 2,6-dimethylaniline and phenyl isocyanate. Yield: quantitative (white solid): mp 240–241 °C; LC/MS (70 eV) *m*/*z* (%): 263 [M^+^, +23]; IR (KBr): 3287 (NH), 1633 (C=O) cm^−1^; ^1^H NMR (300 MHz, DMSO-*d*_6_): δ 2.18 (s, 6H, 2CH_3_), 6.91 (d, *J* = 7.0 Hz, 1H, Ar), 7.04 (s, 3H, Ar), 7.23 (t, *J* = 7.9 Hz, 2H, Ar), 7.43 (d, *J* = 7.6 Hz, 2H, Ar), 7.69 (s, 1H, NH, exch. D_2_O), 8.72 (s, 1H, NH, exch. D_2_O); ^13^C NMR (75 MHz, DMSO-*d*_6_): δ 18.7 (2C), 118.3 (2C), 121.8 (2C), 126.4 (1C), 128.1 (2C), 129.1 (2C), 135.8 (1C), 136.0 (1C), 140.8 (1C), 153.6 (1C). Anal. calcd for C_15_H_16_N_2_O (240.12)%: C 74.97; H 6.71; N 11.66. Found%: C 75.41; H 6.60; N 11.80.

*1-(4-Methoxyphenyl)-3-phenylurea *(**1df**) [38]. Prepared from phenyl isocyanate and *p*-anisidine. Yield: 64% (white needle-shaped fine crystals): mp 216–218 °C; LC/MS (70 eV) *m*/*z* (%): 265 [M^+^, +23]; IR (KBr): 3288 (NH), 1633 (C=O) cm^−1^; ^1^H NMR (300 MHz, DMSO-*d*_6_): δ 3.69 (s, 3H, O-CH_3_), 6.85 (d, *J* = 8.8 Hz, 2H, Ar), 6.93 (t, *J* = 7.0 Hz, 1H, Ar), 7.24 (t, *J* = 7.6 Hz, 2H, Ar), 7.34 (d, *J* = 8.8 Hz, 2H, Ar), 7.42 (d, *J* = 8.2 Hz, 2H, Ar), 8.44 (s, 1H, NH, exch. D_2_O), 8.55 (s, 1H, NH, exch. D_2_O); ^13^C NMR (125 MHz, DMSO-*d*_6_): δ 55.6 (1C), 114.4 (2C), 118.5 (2C), 120.4 (2C), 122 (1C), 129.2 (2C), 133.2 (1C), 140.3 (1C), 153.2 (1C), 154.9 (1C). Anal. calcd for C_14_H_14_N_2_O_2_·0.30 H_2_O (248.27)%: C 67.73; H 5.95; N 11.28. Found %: C 67.43; H 5.76; N 11.14.

*1-(2-Chlorophenyl)-3-(2-methylphenyl)urea *(**1gh**). Prepared from *o*-chloroaniline and *o*-tolyl isocyanate. Yield: 87% (off-white crystals): mp 223–224 °C (acetone/CHCl_3_); LC/MS (70 eV) *m*/*z* (%): 283 [M^+^, +23]; IR (KBr): 3284 (NH), 1643 (C=O) cm^−1^; ^1^H NMR (300 MHz, DMSO-*d*_6_): δ 3.33 (s, 3H, CH_3_), 6.96–7.03 (m, 2H, Ar), 7.11–7.18 (m, 2H, Ar), 7.24–7.30 (m, 1H, Ar), 7.44 (q, *J* = 3.2 Hz, 1H, Ar), 7.75 (d, *J* = 8.2 Hz, 1H, Ar), 8.11 (d, *J* = 1.7 Hz, 1H, Ar), 8.63 (d, *J* =12.4 Hz, 2H, NH exch. D_2_O); ^13^C NMR (125 MHz, DMSO-*d*_6_): 18.3 (1C), 121.1 (1C), 122.2 (1C), 123.4 (1C), 123.5 (1C), 126.3 (2C), 127.7 (1C), 128.5 (1C), 129.4 (1C), 130.5 (1C), 136.4 (1C), 137.2 (1C), 152.7 (1C). Anal. calcd for C_14_H_13_N_2_OCl (260.07)%: C 64.49; H 5.03; N 10.74. Found%: C 64.20; H 4.97; N 10.68.

*1-(3,4-Dichlorophenyl)-3-phenylurea *(**1ef**) [39]. Prepared from 3,4-dichloroaniline and phenyl isocyanate. Yield: 76% (slightly greenish crystals): mp 215–216 °C (acetone/CHCl_3_); LC/MS (70 eV) *m*/*z* (%):280 [M^+^, –1]; IR (KBr): 3319 (NH), 1594 (C=O) cm^−1^; ^1^H NMR (300 MHz, DMSO-*d*_6_): δ 6.97 (t, *J* = 7.3 Hz, 1H, Ar), 7.20–7.35 (m, 3H, Ar), 7.40–7.52 (m, 3H, Ar), 7.86 (d, *J* = 2.3 Hz, 1H, Ar), 8.77 (s, 1H, NH, exch. D_2_O), 8.96 (s, 1H, NH, exch. D_2_O); ^13^C NMR (125 MHz, DMSO-*d*_6_): δ 118.8 (1C), 118.9 (2C), 119.7 (1C), 122.7 (1C), 123.5 (1C), 129.2 (2C), 131.0 (1C), 131.5 (1C), 139.7 (1C), 140.4 (1C), 152.7 (1C). Anal. calcd for C_13_H_10_N_2_OCl_2_·0.25 H_2_O (285.43)%:C 54.66; H 3.71; N 9.81. Found%: C 54.99; H 3.55; N 9.86.

*1-(4-Chlorophenyl)-3-phenylurea (***1cf***, MCC)* [40]. Prepared from 4-CA and phenyl isocyanate. Yield: 90% (white crystals): mp 228–229 °C (acetone/CHCl_3_); LC/MS (70 eV) *m*/*z* (%): 269 [M^+^, +23]; IR (KBr): 3302 (NH), 1636 (C=O) cm^−1^; ^1^H NMR (300 MHz, DMSO-*d*_6_): δ 6.95 (t, *J* = 7.3 Hz, 1H, Ar), 7.23–7.32 (m, 4H, Ar), 7.41–7.49 (m, 4H, Ar), 8.67 (s, 1H, NH, exch. D_2_O), 8.86 (s, 1H, NH, exch. D_2_O); ^13^C NMR (125 MHz, DMSO-*d*_6_): δ 118.8 (2C), 120.2 (2C), 122.4 (1C), 125.8 (2C), 129.0 (1C), 129.2 (2C), 139.2 (1C), 139.9 (1C), 152.9 (1C). Anal. calcd for C_13_H_11_N_2_OCl (246.58)%:C 63.29; H 4.49; N 11.36. Found%: C 63.67; H 4.50; N 11.40.

*1-(4-Chlorophenyl)-3-(4-chlorophenyl)urea (***1cc***, DCC)* [41]. Prepared from 4-CA and 4-chlorophenyl isocyanate. Yield: 86% (pinkish solid): mp 223–224 °C; LC/MS (70 eV) *m*/*z* (%): 281 [M^+^]; IR (KBr): 3296 (NH), 1651 (C=O) cm^−1^; ^1^H NMR spectrum was in agreement with the literature [42]. Anal. calcd for C_13_H_10_ClN_2_O·0.25 H_2_O (285.64)%: C 54.66; H 3.71; N 9.81. Found%: C 54.85; H 3.51; N 9.80.

*1-(3,4-Dichlorophenyl)-3-(2-methylphenyl)urea *(**1eg**). Prepared from 3,4-dichloroaniline and *o*-tolyl isocyanate. Yield: 81% (white needle-shaped fine crystals): mp 217–218 °C (acetone/CHCl_3_/MeOH); LC/MS (70 eV) *m*/*z* (%): 295 [M^+^]; IR (KBr): 3294 (NH), 1643 (C=O) cm^−1^; ^1^H NMR (500 MHz, DMSO-*d*_6_): δ 3.32 (s, 3H, CH_3_), 6.96 (t, *J* = 7.8 Hz, 1H, Ar), 7.12–7.17 (m, 2H, Ar), 7.27–7.29 (m, 1H, Ar), 7.49 (d, *J* = 8.8 Hz, 1H, Ar), 7,74 (d, *J* = 7.8 Hz, 1H, Ar), 7.89 (d, *J*= 2.4Hz, 1H, Ar), 8.00 (s, 1H, NH exch. D_2_O), 9.27 (s, 1H, NH exch. D_2_O); ^13^C NMR (125 MHz, DMSO-*d*_6_): δ 18.2 (2C), 118.6 (1C), 119.5 (1C), 122.1 (1C), 123.4 (1C), 126.6 (1C), 128.7 (1C), 130.7 (1C), 131.0 (1C), 131.5 (1C), 137.3 (1C), 140.5 (1C), 152.9 (1C). Anal. calcd for C_14_H_12_Cl_2_N_2_O (295.14)%: C 56.97; H 4.10; N 9.49. Found%: C 57.03; H 4.19; N 9.47.

*1-(3,4-Difluorophenyl)-3-(2-methylphenyl)urea* (**1gj**). Prepared from 3,4-difluoroaniline and *o*-tolyl isocyanate. Yield: 79% (off-white crystals): mp 210–211 °C (acetone/CHCl_3_); LC/MS (70 eV) *m*/*z* (%): 295 [M^+^, +23]; IR (KBr): 3306 (NH), 1632 (C=O) cm^−1^; ^1^H NMR (300 MHz, DMSO-*d*_6_): δ 2.21 (s, 3H, CH_3_), 6.95 (t, *J* = 7.3 Hz, 1H, Ar), 7.05–7.17 (m, 3H, Ar), 7.27–7.37 (m, 1H, Ar), 7.64–7.71 (m, 1H, Ar), 7.76 (d, *J* = 7.6 Hz, 1H, Ar), 7.96 (s, 1H, NH exch. D_2_O), 9.19 (s, 1H, NH exch. D_2_O); ^13^C NMR (125 MHz, DMSO-*d*_6_): δ 18.3 (1C), 107.4 (1C), 114.5 (1C), 117.8 (1C), 121.9 (1C), 126.6 (1C), 123.5 (1C), 128.5 (1C), 130.7 (1C), 137.4 (1C), 137.5 (1C), 145.8 (1C), 148.6 (1C), 153.0 (1C). Anal. calcd for C_14_H_12_N_2_OF_2_·0.20 H_2_O (265.95)%: C 63.25; H 4.70; N 10.57. Found%: C 63.02; H 4.62; N 10.41.

*1-(3-Chloro-4-fluorophenyl)-3-(2-methylphenyl)urea *(**1gk**). Prepared from 3-chloro-4-fluoroaniline and *o*-tolyl isocyanate. Yield: 92% (off-white crystals): mp 210–211 °C (acetone/CHCl_3_); LC/MS (70 eV) *m*/*z* (%): 301 [M^+^, +23]; IR (KBr): 3323 (NH), 1642 (C=O) cm^−1^; ^1^H NMR (300 MHz, DMSO-*d*_6_): δ 3.33 (s, 3H, CH_3_), 6.95 (t, *J* = 7.3 Hz, 1H, Ar), 7.10–7.20 (m, 2H, Ar), 7.22–7.34 (m, 2H, Ar), 7.75 (d, *J* = 8.3 Hz, 1H, Ar), 7.82 (dd, *J* = 6.4, 2.3 Hz, 1H, Ar), 7.96 (s, exch. D_2_O, 1H, NH), 9.17 (s, exch. D_2_O, 1H); ^13^C NMR (125 MHz, DMSO-*d*_6_): δ 16.9 (1C), 113.6 (1C), 114.6 (1C), 120.9 (1C), 123.6 (1C), 124.2 (1C), 125.9 (1C), 129.3 (1C), 131.7 (1C), 131.9 (1C), 134.2 (1C), 135.2 (1C), 152.9 (1C), 154.8 (1C). Anal. calcd for C_14_H_12_N_2_OFCl·0.25 H_2_O (282.56)%: C 59.37; H 4.45; N 9.89. Found%: C 59.62; H 4.36; N 9.84.

*1-(2-Metylphenyl)-3-(2-fluorophenyl)urea *(**1gl**). Prepared from 2-fluoroaniline and *o*-tolyl isocyanate. Yield: quantitative yield (off-grey crystals); mp 224–225 °C (acetone); LC/MS (70 eV) *m*/*z* (%): 267 [M^+^, +23]; IR (KBr): 3290 (NH), 1644 (C=O) cm^−1^; ^1^H NMR (300 MHz, DMSO-*d*_6_): δ 2.24 (s, 3H, CH_3_), 6.91–7.09 (m, 2H, Ar), 7.11–7.25 (m, 4H, Ar), 7.84 (d, *J* = 7.6 Hz, 1H, Ar), 8.18 (dt, *J* = 8.6, 1.5 Hz, 1H, Ar), 8.34 (s, 1H, NH exch. D_2_O), 8.96 (br s, 1H, NH exch. D_2_O); ^13^C NMR (125 MHz, DMSO-*d*6): δ 18.4 (1C), 115.3 (d, *J*CF = 18.9 Hz, 1C), 120.9 (1C), 121.6 (1C), 122.6 (d, *J*CF = 7.5 Hz, 1C), 123.3 (1C), 124.9 (1C), 126.6 (1C), 128.0 (1C), 128.2 (1C), 130.7 (1C), 137.6 (1C), 152.3 (d, *J*CF = 241.3 Hz, 1C), 152.9 (1C). Anal. calcd for C_14_H_12_N_2_OF (244.26)%: C 68.84; H 5.36; N 11.47. Found%: C 68.74; H 5.34; N 11.37.

*1-(3,4-Difluorophenyl)-3-phenylurea *(**1fj**). Prepared from 3,4-difluoroaniline and phenyl isocyanate. Yield: 58% (greyish crystals): mp 213–214 °C (acetone); LC/MS (70 eV) *m*/*z* (%): 271 [M^+^, +23]; IR (KBr): 3298 (NH), 1630 (C=O) cm^−1^; ^1^H NMR (500 MHz, DMSO-*d*_6_): δ 6.87 (t, *J* = 7.6 Hz, 1H, Ar), 7.05–7.15 (m, 1H, Ar), 7.24–7.32 (m, 3H, Ar), 7.42 (d, *J* = 7.3 Hz, 2H, Ar), 7.60–7.70 (m, 1H, Ar), 8.70 (s, exch. D_2_O, 1H, NH), 8.85 (s, 1H, exch. D_2_O, NH); ^13^C NMR (125 MHz, DMSO-*d*_6_): δ 107.6 (d, *J*CF = 21.9 Hz, 1C), 114.8 (dd, *J*CF = 5.6, 3.7 Hz, 1C), 117.8 (d, *J*CF = 1.7 Hz, 1C), 118.9 (1C), 122.5 (1C), 129.2 (1C), 137.3 (dd, *J*CF = 9.5, 2.8 Hz, 1C), 139.8 (1C), 143.8 (d, *J*CF = 12.4 Hz, 1C), 145.8 (d, *J*CF = 12.4 Hz,1C), 148.5 (d, *J*CF = 13.3 Hz, 1C), 150.5 (d, *J*CF = 13.4 Hz, 1C), 152.9 (1C). Anal. calcd for C_13_H_10_N_2_OF_2_ (248.23)%: C 62.90; H 4.06; N 11.29. Found%: C 62.61; H 4.08; N 11.19.

*1-(3,4-Dichlorophenyl)-3-p-tolylurea *(**1ae**) [43]. Prepared from *p*-toluidine and 3,4-dichlorophenyl isocyanate. Yield: 21% (white fine spicules crystals): mp 225–226 °C (acetone/CHCl_3_); LC/MS (70 eV) *m*/*z* (%): 317 [M^+^, +23]; IR (KBr): 3289 (NH), 1633 (C=O) cm^−1^; ^1^H NMR (500 MHz, DMSO-*d*_6_): δ 2.06 (s, 3H, CH_3_), 7.07 (d, *J* = 8.3Hz, 2H, Ar), 7.28–7.32 (m, 3H, Ar), 7.48 (d, *J* = 8.8 Hz, 1H, Ar), 7.86 (d, *J* = 2.4 Hz, 1H, Ar), 8.65 (s, exch. D_2_O, 1H, NH), 8.91 (s, exch. D_2_O, 1H, NH); ^13^C NMR (125 MHz, DMSO-*d*6): δ 20.8 (1C), 118.7 (1C), 119.1 (1C), 119.6 (1C), 123.4 (2C), 129.6 (2C), 130.9 (1C), 131.5 (1C), 131.6 (1C), 137.1 (1C), 140.5 (1C), 152.8 (1C). Anal. calcd for C_14_H_12_C_l2_N_2_O (294.16)%: C 56.97; H 4.10; N 9.49. Found%: C 56.56; H 4.09; N 9.36.

*1-(3,4-Dichlorophenyl)-3-(4-methoxyl)phenylurea *(**1de**) [44]. Prepared from 3,4-dichloroaniline and *p*-methoxylphenyl isocyanate. Yield: 97% (greyish crystals): mp 234–235 °C (acetone); LC/MS (70 eV) *m*/*z* (%): 333 [M^+^, +23]; IR (KBr): 3263 (NH), 1644 (C=O) cm^–1^; ^1^H NMR (300 MHz, DMSO-*d*_6_): δ 3.69 (s, 3H, CH_3_), 6.82–6.88 (m, 2H, Ar), 7.27–7.36 (m, 3H, Ar), 7.47 (d, *J* = 8.8 Hz, 1H, Ar), 7.84 (d, *J* = 2.3 Hz, 1H, Ar), 8.58 (s, 1H, NH), 8.89 (s, 1H, NH); ^13^C NMR (125 MHz, DMSO-*d*_6_): δ 55.6 (1C), 114.4 (2C), 119.6 (1C), 120.4 (1C), 120.9 (2C), 123.4 (1C), 130.9 (1C), 131.5 (2C), 132.4 (1C), 140.4 (1C), 152.8 (1C), 155.2 (1C). Anal. calcd for C_14_H_12_N_2_O_2_C_l2_ (311.16)%: C 54.04; H 3.89; N 9.00. Found%: C 54.17; H 3.95; N 8.94.

### 3.2. Antibacterial In Vitro Evaluation 

MICs (μg/mL) were determined by the broth microdilution method, using 96-well plates, according to CLSI [26]. Stock solutions of the investigated compounds were obtained by setting the concentration at the maximum possible value. Then, the stock solutions were diluted 1:10 with Mueller Hinton Broth (Oxoid, Italy). Afterwards, twofold serial dilutions in the suitable test medium were carried out to obtain a set of concentrations from 512 to 2 µg/mL in the wells. To obtain the stock solution, DMSO (100%) was employed as diluent. The following bacteria strains, available as freeze-dried discs, belonging to the ATCC collection, were used: Gram-positive: *S. aureus* ATCC 29213, 6538, and 6538P, *E. faecalis* ATCC 29212; Gram-negative: *K. pneuomoniae* ATCC 13883, *P. aeruginosa* ATCC 27853. To conserve the purity of cultures and to enable their reproducibility, cryovials of all microbial strains in the medium were set up and stored at −80 °C. Pre-cultures of each bacterial strain were prepared in Mueller Hinton Broth (MHB) and incubated at 37 °C for 3–5 h. The turbidity of bacterial cell suspension was calibrated to 0.5 McFarland Standard by the spectrophotometric method (OD 625 nm 0.08–0.10), as indicated in the CLSI protocol M7-A9 and, further, the standardized suspension was diluted (1:100) with MHB to reach 1–2 × 10^6^ CFU/mL. All wells were seeded with 100 µl of the mentioned final inoculum, and some wells contained only inoculated broth as control growth. The plates were incubated at 37 °C for 24 h, and the MIC values were recorded as the lowest concentration of compounds at which there was no optically detectable microorganism growth and evaluated by comparing the growth in every well visually with that of the growth control well for bacteria. The MICs were determined by using the assay repeated twice in triplicate. Throughout the study, norfloxacin was used as reference antibiotic. Appropriate controls involved norfloxacin for bacteria as international reference standard. Additionally, DMSO alone (used as diluent of tested compounds) was tested for its activity against bacteria, and consequently, quantities of this solvent under 5% concentration were used during the execution of the experiments. Each species was tested at least three times in duplicate. The MBM assay for bacteria was based on the recommended procedures by CLSI [26].

### 3.3. Cell Cultures

Human MCF-7 and MDA-MB-231 breast cancer cells, HeLa and ISHIKAWA uterine carcinoma cells, A2058 melanoma epithelial cells, MCF-10A mammary epithelial cells and Hek-293 embryonic kidney epithelial cells were obtained from American Type Culture Collection (ATCC, Manassas, VA, USA). Estrogen receptor positive [(ER(+)] MCF-7 and triple negative MDA-MB-231 breast cancer cells were maintained in Dulbecco’s modified Eagle’s medium/nutrient mixture F-12 Ham (DMEM-F12) medium containing 2 mmol/L L-glutamine, 1 mg/mL penicillin-streptomycin and supplemented with 5% fetal bovine serum (FBS) [45]. HeLa epithelial cervix carcinoma cells, estrogen receptor negative [ER(-)], and Ishikawa endometrial adenocarcinoma cell, estrogen receptor positive [ER(+)], were kept in minimum essential medium (MEM), supplemented with 10% FBS, 100 U/mL penicillin/streptomycin and 1% non-essential amino acid. A2058 highly metastatic melanoma epithelial cells were kept in DMEM low glucose (1 g/L), supplemented with 20% FBS, 1% l-glutamine and 100 U/mL penicillin/streptomycin. MCF-10A mammary epithelial cells, were cultured in DMEM/F12 medium, supplemented with 5% horse serum (HS), 100 U/mL penicillin/streptomycin, 0.5 mg/mL hydrocortisone, 20 ng/mL human epidermal growth factor (hEGF), 10 mg/mL insulin and 0.1 mg/mL cholera enterotoxin (Sigma-Aldrich, Milan, Italy). Hek-293 embryonic kidney epithelial cells were cultured in DMEM high glucose (4.5 g/L), supplemented with 10% FBS, 1% l-glutamine and 100 U/mL penicillin/streptomycin. Cells were conserved at 37 °C in a humid atmosphere of 95% air and 5% CO_2_, and screened for contamination at regular intervals [46,47].

### 3.4. Cell Viability

Cell viability was determined using the 3-(4,5-dimethylthiazol-2-y1)-2,5-diphenyltetrazolium bromide (MTT, Sigma-Aldrich, Milan, Italy) assay [48,49]. Cells were seeded on 48-well plates and grown in complete medium. Before treatment, cells were starved in serum free medium for 24 h to allow cell cycle synchronization. Then, cells were treated in phenol-red-free medium supplemented with 1% of serum with increasing concentrations of each compound for 72 h; then, fresh MTT, re-suspended in phosphate-buffer saline (PBS), was added to each well (final concentration (0.5 mg/mL). After 2 h incubation at 37 °C, cells were lysed with DMSO, and then optical density was measured at 570 nm using a microplate reader. For each sample, the mean absorbance was expressed as a percentage over the control and plotted versus drug concentrations to determine the IC_50_ values (i.e., drug concentrations able to decrease cell viability by 50% with respect to control) for each cell line, using GraphPad Prism 8 software (GraphPad Inc., San Diego, CA, USA). Data are representative of three independent experiments; standard deviations (SD) are shown.

## 4. Conclusions

In the search for new compounds endowed with antibacterial activity but with more favorable toxicological properties than TCC, a series of diarylureas were synthesized. Structure–activity relationship studies showed that the 3,4-disubstitution pattern of TCC seems to be not essential for antibacterial activity. Indeed, the highest antimicrobial activity against *S. aureus* was found for compounds bearing a 2,6-xylyl moiety. Particularly, compounds **1ab** and **1bc** were the most interesting of the series showing the same activity as TCC against *S. aureus* and an even higher activity than TCC against *E. faecalis*. These compounds also displayed no cytotoxicity against MCF-10A and Hek-293 cell lines. Based on their simple preparation and interesting antibacterial activity profile, the newly prepared small molecules are promising candidates for antibacterial drug development to be introduced in personal care products in substitution of TCC.

## Figures and Tables

**Table 1:** Physicochemical properties and antibacterial activity results of diarylureas.

			Microorganisms (MIC, µg/mL)						
			Gram-Positive				Gram-Negative		
**COMPD**	**clog*P*^a^**	**milog*P*^b^**	***S.a.* 29213**	***S.a.* 6538**	***S.a.* 6538P**	***E.f.* 29212**	***E.c.*** **25922**	***P. a.* 27853**	***K.p.* 13883**
**1ab**	4.24 ± 0.30	3.46	>256	16	16	32	>256	>256	>256
**1bc**	4.77 ± 0.33	3.69	256	16	16	32	>256	>256	>256
**1ad**	3.27 ± 0.32	3.65	128	32	32	128	>256	>256	>256
**1bd**	3.73 ± 0.32	3.06	>256	32	16	128	>256	>256	>256
**1be**	5.68 ± 0.36	4.29	128	32	128	64	>256	>256	>256
**1af**	3.32 ± 0.29	3.59	128	64	128	64	>256	>256	>256
**1ag**	3.78 ± 0.30	3.99	256	128	256	128	>256	>256	>256
**1ce (TCC)**	5.75 ± 0.38	5.11	16	128	16	64	256	256	256
**1ff (NCC)**	2.86 ± 0.29	3.14	>256	128	64	>256	>256	>256	>256
**1fg**	3.32 ± 0.29	3.54	256	32	16	64	>256	>256	256
**1bf**	3.78 ± 0.30	3.01	>256	16	16	64	>256	>256	>256
**1df**	2.81 ± 0.31	3.20	256	64	128	128	>256	256	>256
**1gh**	3.83 ± 0.32	4.17	256	32	32	256	>256	>256	>256
**1ef**	4.75 ± 0.35	4.43	256	16	128	128	>256	256	>256
**1cf (MCC)**	3.85 ± 0.32	3.82	128	64	128	64	>256	>256	>256
**1cc (DCC)**	4.84 ± 0.35	4.50	256	128	256	128	>256	>256	>256
**1eg**	5.22 ± 0.35	4.83	256	64	128	64	>256	>256	>256
**1gj**	4.16 ± 0.52	3.80	>256	64	128	64	>256	>256	>256
**1gk**	4.91 ± 0.45	4.31	256	128	64	64	-	256	256
**1gl**	3.28 ± 0.40	3.66	128	64	32	128	>256	>256	>256
**1fj**	3.70 ± 0.51	3.40	256	128	64	128	-	256	256
**1ae**	5.22 ± 0.35	4.88	128	64	64	128	>256	>256	>256
**1de**	4.70 ± 0.37	4.48	128	64	64	64	>256	>256	>256
**NRF**	-	-	0.5–2	-	-	2–8	0.03–0.12	1–4	0.5–1

Antibacterial activity was assessed using the CLSI assay (2012) [26]. Abbreviations: *S.a.*: *S. aureus*; *E.f.*: *E. faecalis*; *E.c.*: *E. coli*; *P.a.*: *P. aeruginosa*; *K.p.*: *K. pneumoniae*. NRF: norfloxacin; nt: not tested; ^a^ clog*P* values were computed with ACD Labs/Log P and ACD/ChemSketch 2012 from Advanced Chemistry Development (Toronto, Canada); ^b^ milog*P* values were obtained from Molinspiration property calculation service FAQ, https://www.molinspiration.com/cgi-bin/properties (accessed on 2 February 2021); values according to CLSI (2012) [26].

**Table 2:** IC_50_ values (μg/mL) of compounds **1ab**, **1bc** and TCC on human breast, uterine and melanoma cancer cells as determined by using the MTT assay. IC_50_ values are the mean ± SD of three independent experiments performed in triplicate.

Cell Lines	IC_50_ (μg/mL)		
	**1ab**	**1bc**	**1ce (TCC)**
MDA-MB-231	>200	>200	0.89 ± 0.8
MCF-7	>200	>200	0.64 ± 0.5
HeLa	>200	>200	1.01 ± 1.1
Ishikawa	>200	>200	1.68 ± 0.9
A2058	>200	>200	0.89 ± 0.7
MCF-10A	>200	>200	1.43 ± 0.7
Hek-293	>200	>200	1.60 ± 0.8

## Data Availability

The data presented in this study are available in manuscript.

## Abbreviations

4-CA:4-Chloroaniline; CEC, Contaminant of Emerging Concern; CLSI, Clinical and Laboratory Standards Institute; DCC, 4,4′-Dichlorocarbanilide; DMSO, Dimethyl sulfoxide; hEGF, Human epidermal growth factor; FBS, Fetal bovine serum; HS, Horse serum; IR, Infrared; LC-MS, Liquid chromatography-mass spectrometry; MCC, 1-(3-Chlorophenyl)-3-phenylurea; MICs, Minimum Inhibitory Concentrations; MEM, Minimum essential medium; MTT, 3-(4,5-Dimethylthiazol-2-y1)-2,5-diphenyltetrazolium bromide; NCC, Carbanilide; NMR, Nuclear Magnetic Resonance; SD, Standard deviations; TCC, Triclocarban.
